# Obscure Cerebral Disease

**Published:** 1874-03

**Authors:** N. S. Davis


### MEDICAL EXAMINER.

A

*Semi-Monthly Journal of Medical Sciences.*

EDITED BY N. S. DAVIS, M.D., AND F. H. DAVIS, M.D.

No. V. CHICAGO, MARCH 1, 1874. VOL. XV.

### Original Communications.

### OBSCURE CEREBRAL DISEASE.

CLINICAL CASES IN THE MEDICAL WARDS OF MERCY HOSPITAL. SERVICE PROFESSOR N. S. DAVIS.

AMONG the cases occupying the clinical class in the medical wards of the Mercy Hospital during the last two weeks, we select the following, as possessing features of special interest:

CASE I.—This is a laboring-man; aged about forty years; native of Ireland. He was admitted to this hospital eight or ten days since. He had previously been in the county hospital for a considerable time, and had complained of ill-health several months. At the time of his admission here, his countenance wore a dull, dejected expression; the surface was pale and cool; pulse soft, regular, and 75 per minute; respiration regular, but less full than natural; tongue clean; appetite variable; and urine natural.

When first visited, after his admission, he was lying in bed, on his back, with limbs extended, motionless, and apparently unable to speak. After repeated questions, and some shaking, he uttered, slowly, a few words. It appeared, from what could be gathered of his history from his friends, that, several months since, he began to complain of an almost constant pain in the region of the occiput and posterior fontenelle, with depression of spirits, mental apprehension, and, often, sleeplessness at night. These symptoms increased, until he was wholly unable to work, and was sent to the county hospital for treatment. In addition to constant pain in the posterior part of his head and mental depression, he would have periods, lasting from an hour to a whole day, in which he would neither move nor talk, nor pay any heed to the fullness of his bladder, as though he was in a profound stupor; and yet, his respiration and circulation continued quiet, and he evidently heard much that was said to him. Almost every night he had one or more paroxysms of suddenly crying out with barking, or very unusual sounds, ending in alternately opening wide the mouth, and shutting it, with a few rapid protrusions of the tongue. These paroxysms generally lasted but a few minutes. He also complained of frequently seeing objects or persons in the room, and feeling the apprehension that the latter were coming to injure him. He was disposed to remain in bed; and, when induced to get up, his muscular movements were slightly unsteady, with occasional sudden contractions. There was some hyperæsthesia of the scalp, over the seat of pain in his head; and the pupil of the left eye was one-third larger than that of the right.

The patient had been accustomed, for years, before getting sick, to more or less use of alcoholic drinks, and tobacco. The pathology of the case was conceded to be obscure.

But the pain in the upper and posterior part of the head, the dilatation of the left pupil, the paroxysms of irregular muscular motion in the extremities, etc., suggested the opinion that there was a low grade of inflammation or irritation in the membranes covering the cerebellum, and extending to the tubercula quadrigemina. This suggested an alterative and mildly anodyne treatment. He was required to avoid all stimulating drinks; live on milk and farinaceous diet; and to take a teaspoonful of the following prescription each morning, noon, and tea-time :

℞.—Iodide of sodium, ʒ iij.
Bichlor. hydrarg, gr. j.
Fl. ext. conium, ℥ ss.
Syrup of wild cherry, ℥ ij.
Water, ℥ iss.—Mix.

He was also directed to take from fifteen to twenty grains of bromide of potassium, at bed-time, to secure better sleep.

He has now been under the influence of this treatment about ten days, and is certainly much improved. He complains of much less pain in his head; sleeps better at night; and his periods of mental abstraction and irregular muscular movements are less frequent and less severe; there is also a little less dilatation of the pupil of the left eye. The same treatment was continued.*

CASE II.—This is a boy, aged ten years, who has been brought in from the country, and is presented to the clinical class by the request of his attending physician.

*History.*— Up to the time he was eight years of age (two years since) he is represented to have been a healthy, robust boy, physically, and bright and active mentally.

About two years since, while attending school, he began to exhibit certain nervous symptoms, or singular mental traits, that soon developed into incoherent laughing, which, after a few days, was followed by equally incoherent or uncontrollable

* NOTE.—The patient has now been under the above treatment for five or six weeks, and is up, going in and out, with the appearance of being well, although he still has spells of strange feelings, and fearful apprehensions, especially in the night.

crying, with wringing of the hands, pulling and twisting of the clothes, etc. These symptoms were followed by some irregular muscular movements and facial contortions, resembling slight chorea, which still continue.

After a few weeks, he was found to have, almost every night, one or more paroxysms of suddenly crying out, with strange sounds, and muscular contortions, though not like ordinary spasms or convulsions, and not followed by any period of unconsciousness. About two months after the boy was noticed to be unwell, a slight swelling was discovered by his attending physician, in the right inguinal region, which, the boy said, was tender, and sometimes painful; and the doctor found that pressure with the finger, on the swelling, uniformly caused symptoms similar to his nightly paroxysms. After the use of some alterative and cathartic remedies, this swelling, and all local symptoms in the inguinal region, disappeared. The nightly paroxysms, however, have continued, with gradually increasing frequency, until, at present, they average six or seven each night. They consist in starting suddenly from sleep, with crying out, and, generally, violent swinging out of his arms, kicking with the feet; sometimes bending the body forward, followed by violent extension, and reckless tossing, but no frothing at the mouth, stertorous breathing, or stupor. The individual paroxysms last but a few minutes, at most; and the boy retains a good appetite—a good degree of general nutrition; but his gait, in walking, has become awkward, his nervous system impaired, with little power to fix his attention, and little apparent inclination to talk. He is very restless during the day, moving about almost constantly, but to no definite purpose. His bowels are nearly regular; but there occurs, about once in six or seven days, a mucous or slimy discharge, as though there was still some point of irritation in the mucous membrane of the colon.

*Pathology.*—From the preceding history, it will be conceded that the nature of this case is somewhat obscure. As is usual in such cases, the boy has been treated by several medical men, and subjected to a great variety of medication. The gradual impairment of his mental faculties, and altered muscular movements, with the nightly paroxysms, are sufficient to show, not only a morbid condition of the brain, but that long continuance of such irritation or condition, has induced morbid or defective nutrition of the cerebral substance; and, unless it can be removed, it will end in arrest of cerebral growth, and dementia. In its commencement, the grade of morbid action appeared to be intermediate between that of chorea and epilepsy; but, in its progress, it has approximated more and more to the latter disease.

If this view of its pathology be correct, the question would still arise, whether the cerebral irritation was primary, or reflex from some point of disease elsewhere, more especially in the cæcum, or some part of the ileocæcal junction, as suggested by the swelling and tenderness discovered over that region, about two months after the boy began to complain. As a general rule, reflex irritation, in the nervous-centers, does not lead to as marked evidences of impairment of nutrition, and of mental faculties, as is presented in this case; yet, the fact that indications of special local disease, in the right inguinal region, were noticed early in the case, and that there still occurs, every week, a single mucous discharge from the bowels, should not be entirely overlooked.

*Treatment.*—There are three leading ideas that should govern our treatment of the case, in its present stage: one, to remove, as far as practicable, any present existing local disease in the mucous membrane of the intestines; another, to overcome the morbid sensitiveness, or irritation, in the cerebral center; and the other, to restore a healthy, active, nutrition of the brain substance. To meet the first indication, the following prescription was suggested:

℞.—Nit. of silver, gr. x.
Ext. hyoscyamus, gr. xv.—Mix.

Divide into thirty pills, and give one pill before each meal. At the same time, for the second indication, the following was directed:

℞.—Brom. potass., ʒ iv.

Tinct. digitalis, ʒ iv.

Fluid ext. scutellaria, ℥ j.

Syrup wild cherry, ℥ iiss.—Mix.

A teaspoonful to be given half an hour after breakfast, dinner, and at bed-time, in a little water.

The dose, at bed-time, might be increased to a teaspoonful and a half, if found necessary, to interrupt the night paroxysms. The first prescription might be discontinued, after the first two weeks, and a teaspoonful of the compound syrup of the hypophosphites, with an excess of the phosphorous acid, given instead of the pills, which would meet the third indication named. To have a fair chance of success, the second prescription and the hypophosphites should be continued several months, with a diet of milk, farinaceous articles, vegetables, and fruit, but without either *meat or stimulating drinks.* He should be allowed to take a fair amount of out-door exercise, daily, and be subjected to mild, cheerful, mental discipline.

